# Intracoronary Transplantation of Mesenchymal Stem Cells with Overexpressed Integrin-Linked Kinase Improves Cardiac Function in Porcine Myocardial Infarction

**DOI:** 10.1038/srep19155

**Published:** 2016-01-11

**Authors:** Dan Mu, Xin-Lin Zhang, Jun Xie, Hui-Hua Yuan, Kun Wang, Wei Huang, Guan-Nan Li, Jian-Rong Lu, Li-Juan Mao, Lian Wang, Le Cheng, Xiao-Li Mai, Jun Yang, Chuan-Shuai Tian, Li-Na Kang, Rong Gu, Bin Zhu, Biao Xu

**Affiliations:** 1 Department of Cardiology, Affiliated Drum Tower Hospital, Nanjing University School of Medicine, Nanjing, China; 2 Department of Radiology, Affiliated Drum Tower Hospital, Nanjing University School of Medicine, Nanjing, China; 3 Department of Pathology, Affiliated Drum Tower Hospital, Nanjing University School of Medicine, Nanjing, China

## Abstract

The effect of mesenchymal stem cell (MSCs)-based therapy on treating acute myocardial infarction (MI) is limited due to poor engraftment and limited regenerative potential. Here we engineered MSCs with integrin-linked kinase (ILK), a pleiotropic protein critically regulating cell survival, proliferation, differentiation, and angiogenesis. We firstly combined ferumoxytol with poly-L-lysine (PLL), and found this combination promisingly enabled MRI visualization of MSCs *in vitro* and *in vivo* with good safety. We provided visually direct evidence that intracoronary ILK-MSCs had substantially enhanced homing capacity to infarct myocardium in porcine following cardiac catheterization induced MI. Intracoronary transplantation of allogeneic ILK-MSCs, but not vector-MSCs, significantly enhanced global left ventricular ejection fraction (LVEF) by 7.8% compared with baseline, by 10.3% compared with vehicles, and inhibited myocardial remodeling compared with vehicles at 15-day follow-up. Compared with vector-MSCs, ILK-MSCs significantly improved regional LV contractile function, reduced scar size, fibrosis, cell apoptosis, and increased regional myocardial perfusion and cell proliferation. This preclinical study indicates that ILK-engineered MSCs might promote the clinical translation of MSC-based therapy in post-MI patients, and provides evidence that ferumoxytol labeling of cells combined with PLL is feasible in *in vivo* cell tracking.

Despite major advances in pharmacotherapy and revascularization technologies, acute myocardial infarction (MI) remains challenging partially because of post-infarct myocardium remodeling, a process leading to substantial chamber dilation and contractile dysfunction^1^. Regenerative therapies, represented by bone marrow-derived cell transplantation, have emerged as promising novel approaches to address this issue^2^. Bone marrow-derived mesenchymal stem cells (MSCs) show its priority by virtue of great differentiation potential and antifibrotic properties^3^. These beneficial effects of MSCs have been supported by recent preclinical and clinical studies^4^,^5^,^6^,^7^,^8^,^9^, which reveal reduced infarct size and left ventricular (LV) volume, improved regional LV systolic function, or even global LV function. Of note, however, MSCs delivery in these studies were mostly achieved through intramyocardial (epicardial or endocardial) injection, which is either surgically operated or technically demanding^10^. Intracoronary transplantation, which is familiar to interventional cardiologists, gains its popularity because it could be performed during percutaneous intervention (PCI) for acute MI, but the insufficient homing efficacy of stem cells to ischemic myocardium limits its application^11^.

Gene modification could affect the efficacy of MSCs and should not be overlooked^12^. Integrin-linked kinase (ILK), a pleiotropic protein, critically regulates cell survival, proliferation, differentiation, apoptosis and angiogenesis. ILK blockade significantly reduced endothelial progenitor cells (EPCs) homing to ischemic limb^13^, while ILK overexpression significantly enhanced the proliferative, migratory, and angiogenic capabilities of EPC and results in neovascularization^13^,^14^. An effect of inducing cardiomyogenesis of ILK has also been recently documented in human fetal myocardial cells^15^. It’s interesting that ILK expression is absent in endothelium from atherosclerotic arteries^16^, and overexpression of ILK in myocardium results in unequivocally improved LV function and reduced cardiac remodeling after myocardial infarction^17^. Therefore, it’s reasonable and promising to combine these favorable profiles of MSCs and ILK, particularly by enhancing the poor homing capacity and limited regenerative potential of MSCs^18^, through engineering MSCs with ILK to treat acute MI. Indeed, ILK-transfected MSCs have higher survival and adhesion rates *in vitro*, and smaller infarct size than vector-MSCs after intramyocardial injection in a small-animal model of MI^19^.

To accelerate the clinical practice of cell therapy, a deeper understanding of the fate of implanted cells is warranted, which could be partially elucidated through imaging^20^. A body of methods with good safety and efficacy profiles have been developed to track these transplanted cells, by labeling cells with superparamagnetic iron oxide nanoparticles (SPIONs)^21^. However, these SPIONs were removed from market in 2009. Ferumoxytol is a newly FDA-approved ultrasmall superparamagnetic iron oxide (USPIO) in treating iron deficiency anemia in chronic kidney disease^22^. The off-label use of ferumoxytol in labeling stem cells has a good safety, and by together use of magnetic resonance imaging (MRI), a promising capacity for *in vivo* monitoring of implanted cells^23^,^24^.

We postulated that an enhanced homing capacity of MSCs following ILK overexpression could be reached, which could subsequently result in improvements in global cardiac function. We therefore investigated the therapeutic effect of intracoronary-implanted ILK-overexpressing MSCs (ILK-MSCs) on cardiac function in a cardiac-catheterization-induced large-animal model of MI compared with vector-modified MSCs (vector-MSCs) and vehicles (PBS). *In vivo* assessment of myocardial homing of transplanted MSCs was achieved by labeling cells with ferumoxytol, and genetically labeled with green fluorescent protein (GFP) to compensate the limitation of iron-labeling, *i.e.* dilution loss of exogenous labels by cell division. We firstly combined ferumoxytol and poly-L-lysine (PLL) to enhance the capacity of cell labeling. MRI was used to monitor implanted cells^25^ and to determine global and regional LV contractile function, remodeling, scar size and regional myocardial perfusion.

## Results

### ILK Overexpression in MSCs

MSCs from swine bone marrows were isolated and cultured as previously described^26^,^27^. Passages 3 to 4 MSCs were transduced with Ad-ILK-hrGFP at MOI = 5 for 48 hours. Modification efficiency was 90.3% as measured by GFP expression with flow cytometry analysis (Supplementary Fig. 1a,b). Expression of hrGFP in the modified MSCs was confirmed by immunofluorescent assay (Supplementary Fig. 1c), and ILK expression delineated by western blot analysis and quantitative RT-PCR (Supplementary Fig. 1d,e). To verify the effects of ILK on cardiac progenitor cells (CPCs) demonstrated earlier^28^, we evaluated the physiological properties of MSCs following ILK overexpression. Indeed, an increased cell viability, proliferative ability, migration, DNA synthesis, while a decreased cell apoptosis were noted (*P* < 0.001 for all comparisons; Supplementary Fig. 2).

### *In Vitro* Labeling of MSCs with Ferumoxytol

**Figure 1:**
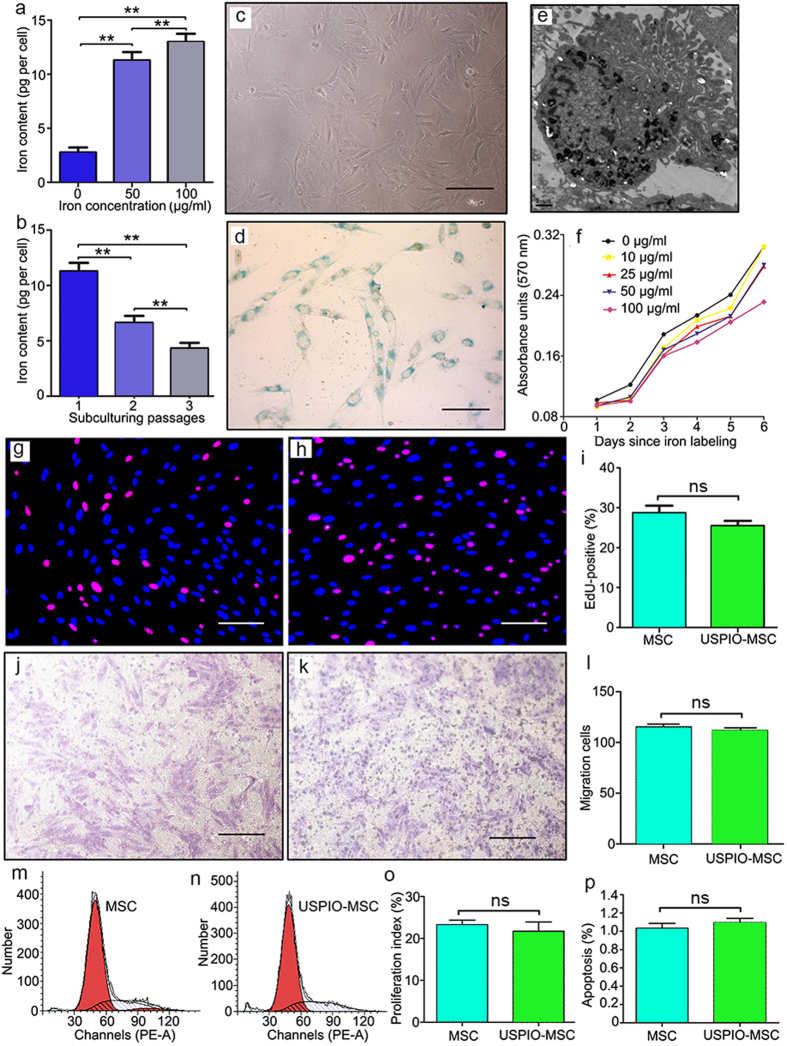
*In vitro* labeling with ferumoxytol does not affect biological properties of swine MSCs. (**a,b**) The ferumoxytol uptake, as assessed by colorimetric ferrozine assay, was proportional to the iron concentration in the culture medium (**a**) and inversely correlated to subculturing passages of MSCs (**b**). (**c,d**) Representative light microscopy images of unstained (**c**) and Prussian-blue-stained (**d**) MSCs. (**e**) Transmission electron microscopy image of iron-labeled MSCs showing iron complex was encapsulated in the endosomes. (**f**) Cell viability of MSCs were tested by 3-(4,5-dimethylthiazol-2-yl)- 2,5-diphenyltetrazolium assay with doses of ferumoxytol up to 100 μg/ml and incubation time up to 72 hours. Error bars from three independent experiments were not shown for better visual effect. (**g–i**) Proliferation of MSCs not labeled or labeled with ferumoxytol was evaluated with 5-ethynyl-2´-deoxyuridine assay. (**j–l**) *In vitro* migration of MSCs not labeled or labeled with ferumoxytol was determined by transwell migration assay. (**m–o**) Cell cycle analysis was performed using propidium iodide staining followed by flow cytometric analysis. Data are mean ± SD. **P < 0.01 between comparisons indicated by bracket; ns = not significant. Scale bar in e, 1.0 μm. Scale bars in others, 10 μm.

Swine MSCs engineered with vector-hrGFP or Ad-ILK-hrGFP were labeled with ferumoxytol at increasing concentrations (up to 100 μg/ml) in the presence of PLL *in vitro*. We observed a linear correlation between ferumoxytol uptake, as assessed by colorimetric ferrozine assay and the iron concentration in the culture medium (Fig. 1a). When MSCs were subcultured for 3–4 passages post-incubation, the iron content in each cell progressively dropped over time, likely because of dilutional loss with cell proliferation (Fig. 1b). Following the third subculturing passage, the iron content per MSC decreased to about 40% of the initial value. Treatment with a dose of 50 μg/ml ferumoxytol for 24 hours resulted in approximately 100% labeling efficacy, evidenced by Prussian blue positive cell rates (Fig. 1c,d). Transmission electron microscopy confirmed that the internalized iron was localized in subcellular structures, likely endosomes (Fig. 1e).

MSCs exposed to a dose of 100 μg/ml of ferumoxytol revealed a significant decrease in cell viability after incubation for 6 days (Fig. 1f). No substantial difference in cell viability was detected at lower doses for the longest incubation time (6 days) or at highest dose before 5 days (Fig. 1f). Therefore, the 50 μg/ml dose was chosen for subsequent experiments, given no significant impairments were documented in cell proliferation, migration, apoptosis or cell cycles at this dose when comparing iron-labeled MSCs to unlabeled controls (Fig. 1g–p).

### *In Vitro* Magnetic Resonance Imaging of Labeled MSCs

**Figure 2:**
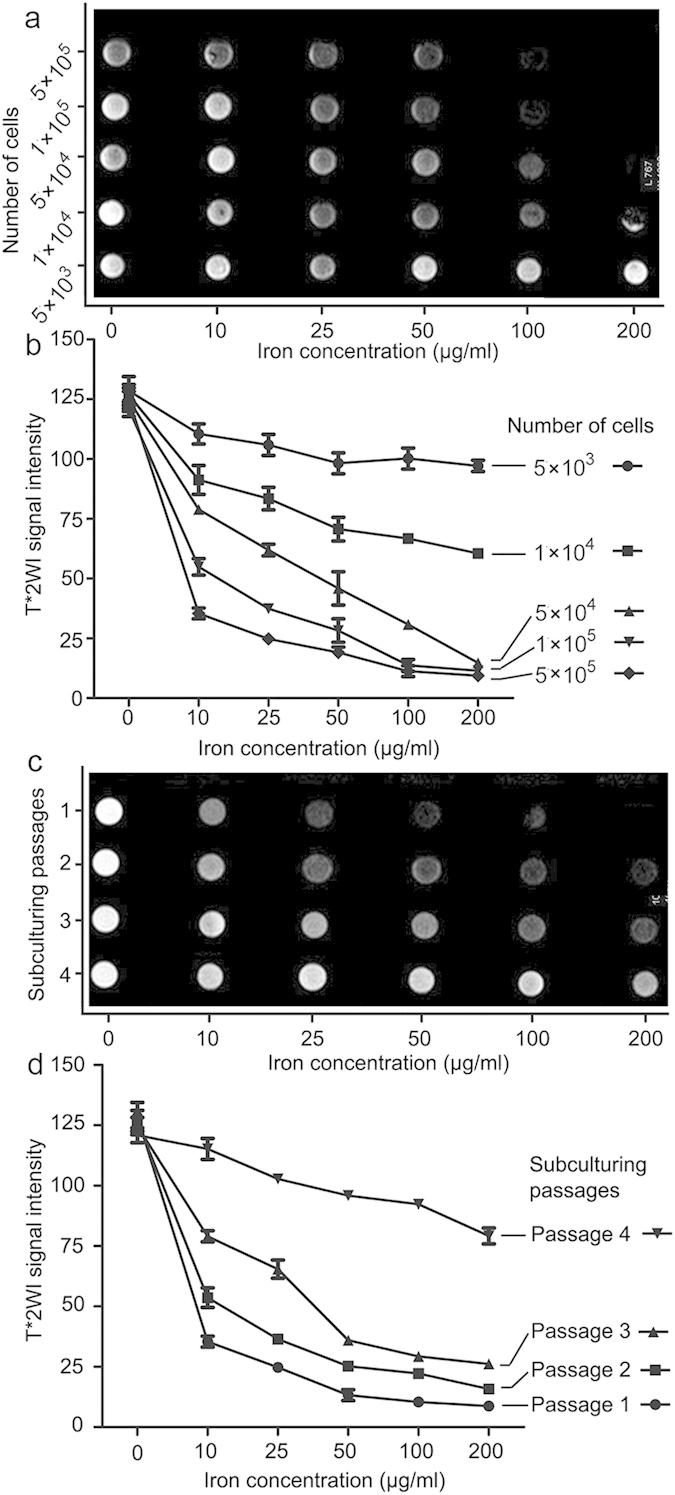
*In vitro* detection of iron-labeled MSCs with MRI. (**a,b**) T2*-weighted MRI were performed using increasing number of MSCs (5 × 10^3^ to 5 × 10^5^) subcultured at increasing concentrations of ferumoxytol (0 to 200 μg/ml). When treating 1 × 10^4^ MSCs with 100 μg/ml ferumoxytol, the hypointense signals from T2*-weighed images were distorted due to blooming effect. (**c,d**) T2*-weighted MRI were performed using of MSCs from different subculturing passages (passage 1 to 4) subcultured at increasing concentrations of ferumoxytol (0 to 200 μg/ml). An inverse correlation was found between negative signal intensity and number of passages. Data are mean ± SD.

The detectability of ferumoxytol-labeled MSCs was tested using MRI. T2*- and T2-weighted MRI were performed using increasing number of MSCs (5 × 10^3^ to 5 × 10^5^) from different subculturing passages (passage 1 to 4) subcultured at increasing concentrations of ferumoxytol (0 to 200 μg/ml) and suspended in low-melting agarose. Good correlations between negative signal intensity and number of MSCs, concentrations of iron supplement and number of passages were observed (Fig. 2a–d). Notably, the number of hypointense voxels decreased remarkably following repeated subculturing of MSCs (Fig. 2c,d). Unlabeled cells were not detected in MR images. When treating 1 × 10^4^ MSCs with 100 μg/ml ferumoxytol, the hypointense signals from T2*-weighed images were distorted due to blooming effect (Fig. 2a), as was similar in T2-weighted images (Supplementary Fig. 3). The potential “false” hypointense signal achieved from a higher dose (100 μg/ml) further reinforced our selection of dose 50 μg/ml, at which T2*-weighted image was able to detect as low as 1 × 10^4^ MSCs, and no obvious blooming effect occurred even treated in 5 × 10^5^ MSCs (Fig. 2a).

### Porcine acute MI model establishment

A total of 20 minipigs were subjected to MI by balloon occlusion of left anterior descending coronary artery beyond the second diagonal branch for 90 minutes after 4 times of preconditioning dilation (Supplementary Fig. 4a,b). Five were excluded before treatment allocation (one death during MRI examination before acute MI model establishment and 4 due to ventricular fibrillation during occlusion procedure). The remaining 15 minipigs were randomly assigned equally to one of the three groups: the vehicle group, vector-MSC group, and ILK-MSC group. Among these, 7 were smoothly operated with only paroxysmal ventricular tachycardia during procedure, and 8 were regarded as flexuous but still successful following repeated defibrillation and pharmacological therapy (Supplementary Fig. 4c–e). MI model were successfully established in all 15 minipigs, as evidenced by substantial ST-segment elevation from electrocardiography monitoring, and late gadolinium enhancement images of infarcted myocardium from MRI scanning. Five healthy non-infarcted minipigs were included, and their cardiac functions were used as references.

### *In Vivo* MRI Tracking and Histological Examination of Labeled MSCs after Transplantation

**Figure 3:**
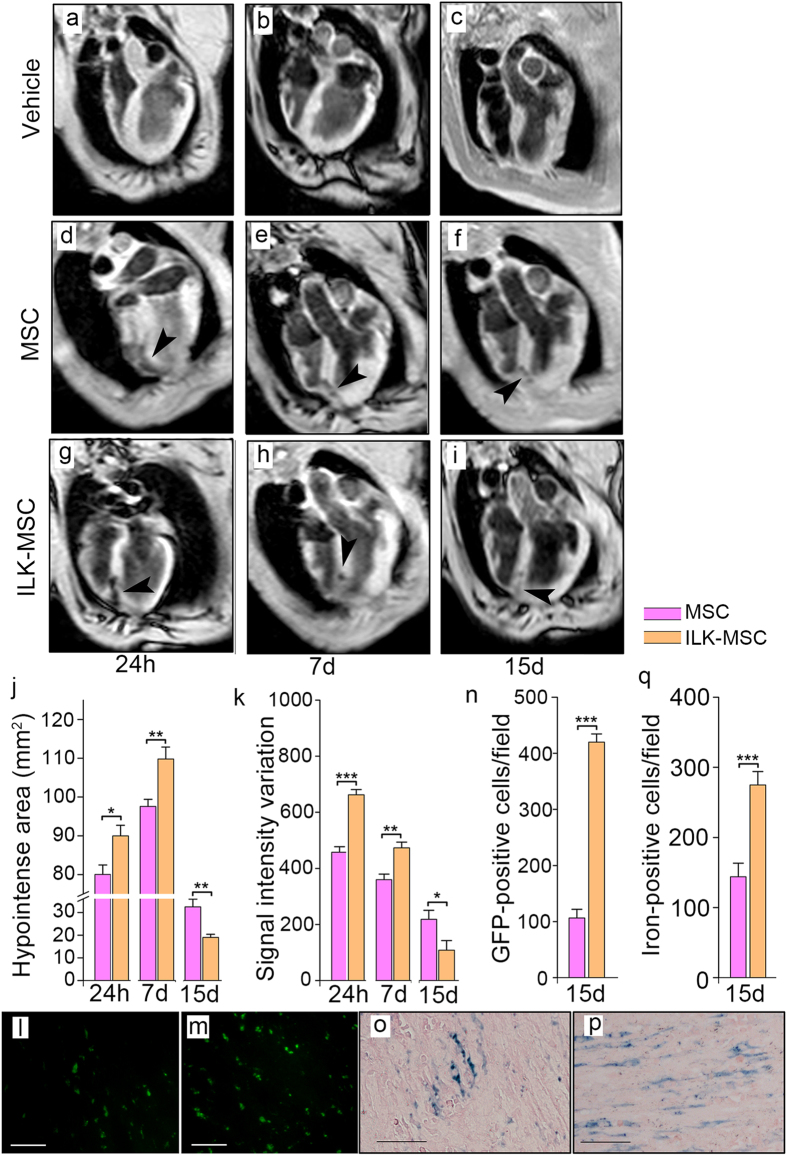
*In vivo* MRI tracking and histological examination of labeled MSCs after transplantation. (**a–l**) Representative T2*-weighted magnetic resonance images of minipig hearts obtained 24 hours, 7 days and 15 days after vehicle control (**a–c**), vector-MSC (**d–f**) and ILK-MSC (**g–i**) transplantation. Black arrow heads indicate hypointense (black) signals associated with iron-labeled MSCs. (**j–k**) Calculated hypointense area (**j**) and signal intensity variation (**k**) corresponding to the magnetic resonance slices in (**d–i**). (**l– n**) Fluorescence microscopy of green fluorescence signal of iron–labeled cells in peri-infarct areas at 15-day follow-up. (**o–q**) Prussian-blue staining of iron-labeled cells in peri-infarct areas at 15-day follow-up, showing that more cells were iron-labeled but iron content per cell was lower in ILK-MSC group than vector-MSC group. Data are mean ± SEM. *P < 0.05, **P < 0.01, ***P < 0.001 between comparisons indicated by bracket. Scale bars, 10 μm.

MSC migration and distribution were monitored with MRI 24 hours after MSC transplantation, as well as 7 days and 15 days post-MSC injection by investigators blinded to treatment assignments. Hypointense signals on T2*-weighed images were detectable 24 hours after transplantation of both vector-MSCs and ILK-MSCs. Significant increases in hypointense signal area were observed 7 days post-injection versus 24 hours (Fig. 3a–k)^29^. Signal intensity variation decreased at 7 days post-transplantation, further decrease occurred in signal intensity variation at 15 days, paralleled with a decrease in hypointense signal area (Fig. 3j–k). The substantial decreasing T2* signal intensity was probably due to cell proliferation^30^.

During the first 7 days post-MSC transplantation, ILK-MSCs exhibited significantly enhanced homing ability to infarcted myocardium compared with vector-MSCs, which could be directly acquired from T2*-weighed images showing larger hypointense area and higher signal intensity variation. It’s interesting that contrary results were observed at 15 days through T2*-weighed images (Fig. 3f,i–k). This may be attributable to higher proliferative ability of ILK-MSCs supported by *in vitro* results that hypointense signals decreased remarkably following repeated subculturing of MSC, and further supported by corresponding histological staining results showing still significantly higher number of GFP- and iron-positive cells at 15 days in peri-infarct areas in ILK-MSC than vector-MSC group (Fig. 3l–q). It is worth noting that iron content per cell in ILK-MSC-treated minipigs was lower than vector-MSC-receiving animals (Fig. 3o,p).

### Global and Regional Left Ventricular Systolic Function Improvement Following ILK-MSCs Transplantation

**Figure 4:**
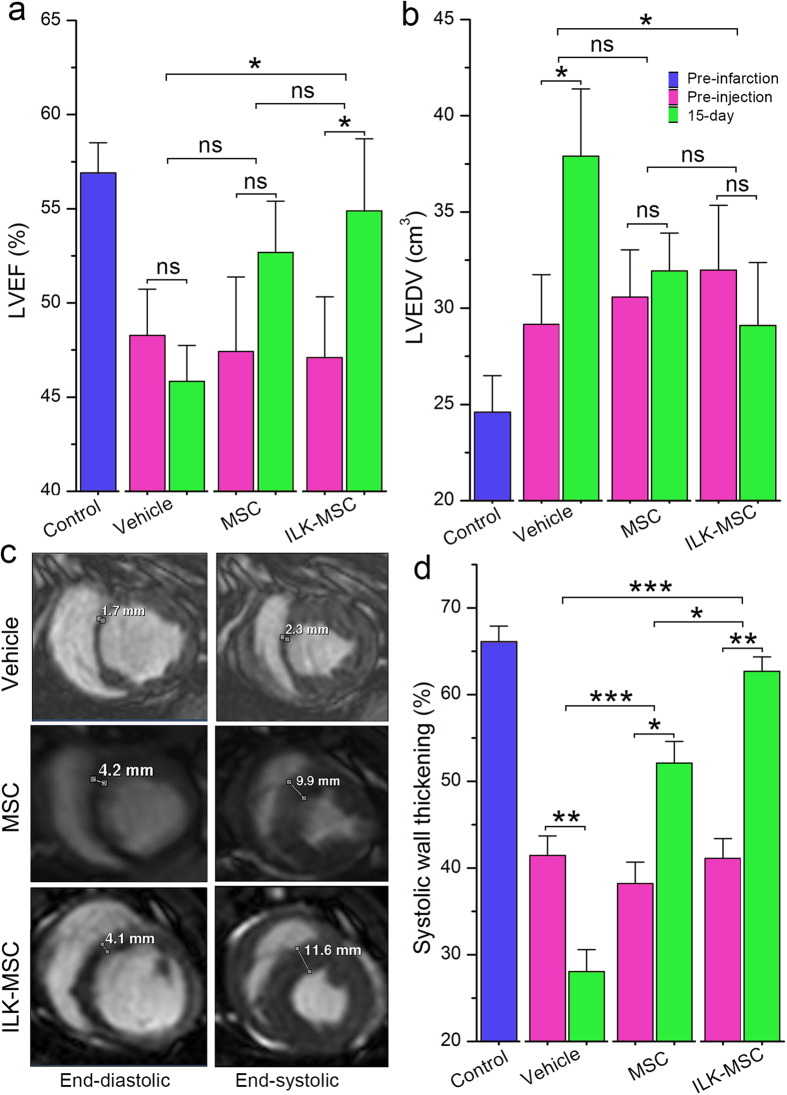
Global and regional left ventricular systolic function improvement following ILK-MSC transplantation. (**a**–**b**) Left ventricular ejection fraction (LVEF, **a**) and left ventricular end-diastolic volume (LVEDV, **b**) at baseline (pre-injection) and 15 days post-transplantation in vehicle controls, vector-MSC group and ILK-MSC group. (**c**) Representative matched cine short-axis images (at end-diastole and end-systole) at 15 days for a minipig treated with vehicles (top row), vector-MSCs (middle row) and ILK-MSCs (bottom row). Infarcted myocardial segments were visually positioned from matched delayed contrast-enhanced images. (**d**) Systolic wall thickening at baseline and 15 days post-transplantation. Data are mean ± SEM. *P < 0.05, **P < 0.01 between comparisons indicated by bracket. ns = not significant.

In terms of global LV function, paired analysis demonstrated a significant improvement of LVEF from baseline to 15 days in ILK-MSCs-receiving minipigs (from 47.1 ± 7.2% at baseline to 54.9 ± 8.6% at 15 days, *P* = 0.03), a nonsignificant increment in vector-MSCs-treated minipigs (from 47.4 ± 8.1% at baseline to 52.7 ± 6.1% at 15 days, *P* = 0.16), and a trend toward decrement in vehicles (from 48.3 ± 5.5% at baseline to 45.8 ± 4.3% at 15 days, *P* = 0.46; Fig. 4a). Accompanied was a significant increasing of left ventricular end-diastolic volume (LVEDV; *P* = 0.01; Fig. 4b) in vehicles, and a nonsignificant trend toward decrease in ILK-MSC-treated group. Analyses of the between-group differences regarding global LVEF at 15 days revealed that treatments with ILK-MSCs (absolute change in LVEF, 7.8% [95% confidence interval (CI): 2.7 to 12.9%]) were associated with significant improvements compared with vehicles (absolute change in LVEF, −2.5% [95% CI: −8.2 to 3.4%]) (*P* = 0.018), but not significant in vector-MSCs (absolute change in LVEF, 5.3% [95% CI: −1.1 to 11.6%]) (*P* = 0.06). In line with the results for LVEF, LVEDV demonstrated a significant decrease in ILK-MSCs group at 15 days (*P* = 0.01), but did not show a significant decrease in vector-MSCs group (*P* = 0.08, Fig. 4b) compared with vehicle PBS.

Regional LV function was evaluated in infarcted myocardium with regional systolic wall thickening. At 15 days, MSC-treated infarcted myocardium displayed improved systolic thickening compared with that of vehicle-treated controls (62.7 ± 3.7% and 52.1 ± 5.6% versus 28.1 ± 5.7%, both *P* < 0.001; Fig. 4c,d). Longitudinal follow-up studies revealed that considerable reduction occurred in control group at 15 days (*P* < 0.01), whereas this natural process was reversed by ILK-MSC and vector-MSC transplants, resulting in substantial recovery of regional function following MSCs implantation (*P* < 0.01 and *P* < 0.05 respectively; Fig. 4c,d). It’s striking that ILK-MSCs achieved significantly higher improvement of regional LV contractile function compared with vector-MSCs (*P* = 0.012; Fig. 4d).

### Infarct Size decrement Following ILK-MSCs Transplantation

**Figure 5:**
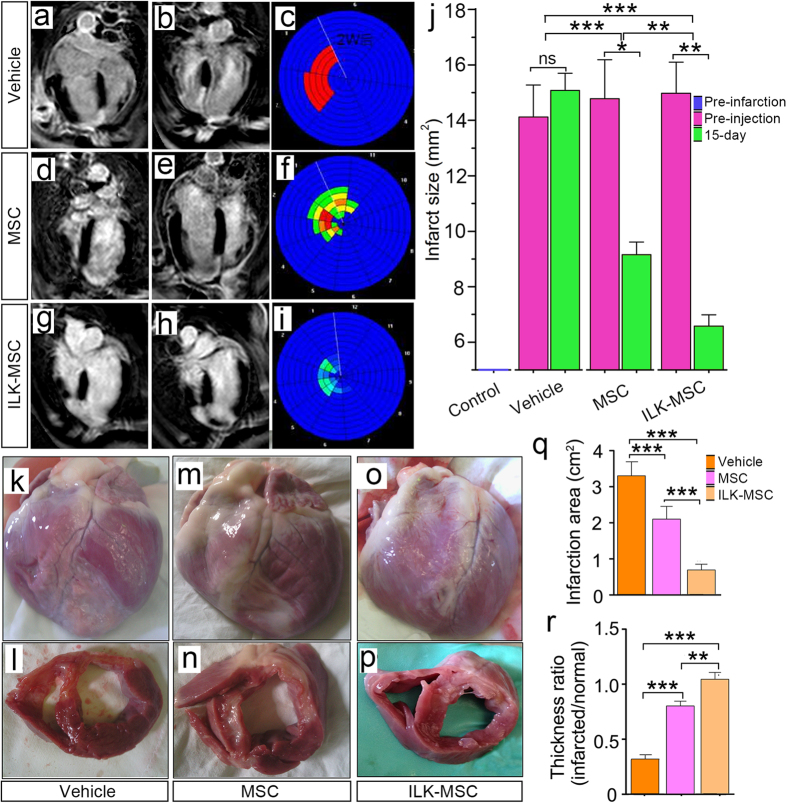
Infarct size decrement following ILK-MSC transplantation. (**a– i**) Representative delayed contrast-enhanced MR images and Bull’s eye plots of hearts in four-chamber view at end-diastole. Infarct scar tissue appears as bright signal whereas viable myocardium appears dark. In vector-MSCs and ILK-MSCs treated minipigs, scar size decreased over the period of 15 days following MSCs transplantation, while in vehicle controls, no evidence of decrement was documented. (**j**) Infarct size at baseline (pre-injection) and 15 days post-transplantation in three groups. Importantly, a significant reduction was noted in ILK-MSC treated minipigs compared with vector-MSC ones. Infarct size was calculated from the short-axis delayed-enhancement images. (**k–p**) Representative macroscopies of entire hearts and short-axis cardiac slices of one vehicle-, one vector-MSC- and one ILK-MSC-treated minipig at 15 days post-implantation. (**q–r**) Calculated infarction area (**q**) and thickness ratio of infarcted myocardium (**r**) corresponding to the macroscopical examinations in (**k–p**). Data are mean ± SEM. *P < 0.05, **P < 0.01, ***P < 0.001 between comparisons indicated by bracket.

The infarcted myocardium was determined by delayed contrast-enhanced imaging. At 15-day follow-up, infarct size in vehicle group increased by 10% compared with initial size, while ILK-MSCs treatment resulted in significantly decreased infarct size with a reduction rate of 56% (*P* < 0.001; Fig. 5a–j). Importantly, the difference between ILK- and vector-MSC treatments was significant (*P* < 0.01). Bull’s eye plots revealed clearer but similar results (Fig. 5c,f,i).

Macroscopical and microscopical examinations further confirmed these findings. Infarct areas of hearts harvested 15 days following transplantation were measured. The infarct area of vehicle minipigs remained large (averaged 3.31 cm^2^), while MSC-treated ones exhibited sizable decrease in infarct size (*P* < 0.001; Fig. 5k–q). Significant difference was also obtained between ILK- and vehicle-MSCs treated minipigs (*P* < 0.001; Fig. 5k–q). The observed reduction in infarct size and paralleled decrement in fibrosis area demonstrated by Masson’s trichrome staining (Supplementary Fig. 5) correlated well with the increment in thickness of the infarcted myocardium (Fig. 5r).

### Regional Myocardial Perfusion Recovery Following ILK-MSCs Transplantation

**Figure 6:**
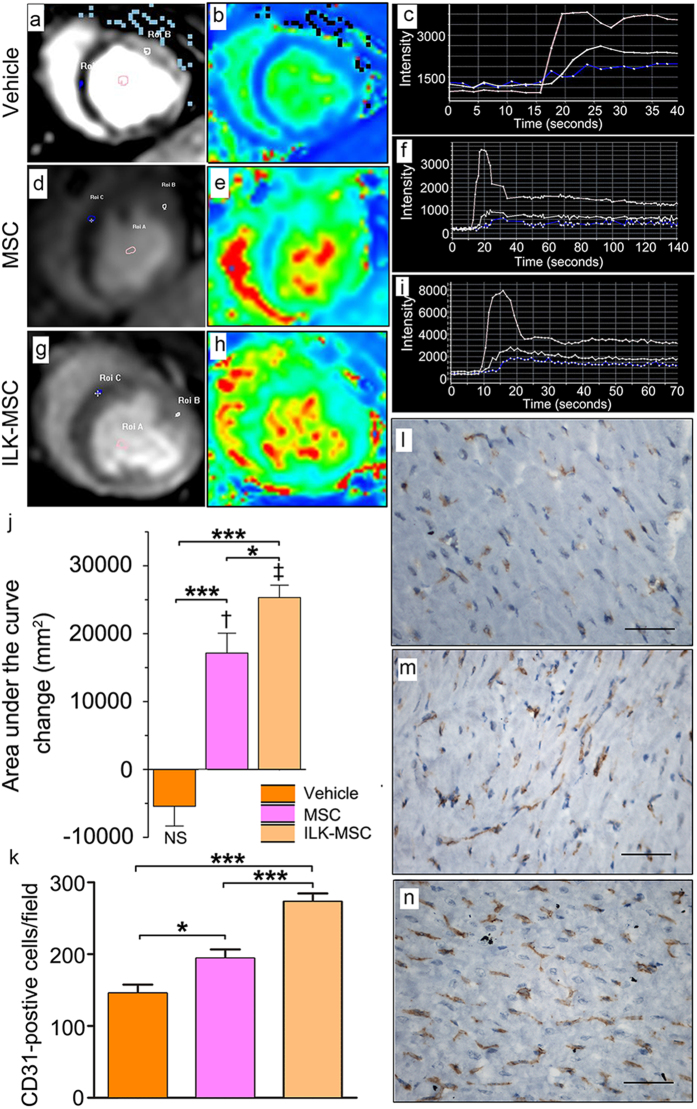
Regional myocardial perfusion recovery following ILK-MSC transplantation. (**a–j**) Representative contrast-enhanced first-pass perfusion images (**a,d,g**) and pseudocolor magnetic resonance images (**b,e,h**) at 15-day follow-up, along with respective time–signal intensity curves (**c,f,i**) from which areas under the curve were calculated (**j**) reflecting blood supply in certain region in one vehicle- (**a–c**), one vector-MSC- (**d–f**) and one ILK-MSC-treated (**g–i**) minipig. Three curves in c indicated blood pool, normal myocardium and infarcted myocardium respectively from top to bottom, as were curves in f and i. Area under the curve changes from baseline to 15 days post-transplantation were calculated, showing significant improvement of regional blood supply in ILK-MSC treated and vector-MSC treated animals, and a substantial difference between these two MSC groups. (**k–n**) Immunohistochemistry staining of CD31 in peri-infarct zones of three groups confirmed an enhanced microvessel density in ILK-MSC treated animals. Data are mean ± SEM. ^†^*P* < 0.01, ^‡^*P* < 0.001 versus baseline; *P < 0.05, ***P < 0.001 between comparisons indicated by bracket. Scale bars in (**l–n**), 10 μm.

Myocardial perfusion was evaluated with first-pass perfusion imaging. Qualitative interpretation was achieved through visual exploration, showing lowest perfusion in vehicle-treated group and higher perfusion in MSC-treated groups (Fig. 6a–i). Time-intensity curves (TICs) were generated and *Integral*, defined as the area under curves (AUC), was computed as parameter reflecting blood supply in certain region^31^. There were significant differences of AUC value from TICs between infarcted and normal myocardium (Fig. 6c,f,i). No difference of AUC in infarcted myocardium was detected across all three groups at baseline (Fig. 6j). Decrement was detected in vehicle group, while progressive perfusion improvement occurred in ILK-MSC- and vector-MSC-treated minipigs at 15-day follow-up (*P* < 0.001 and *P* < 0.01 versus baseline respectively), and the difference between ILK-MSCs and vector-MSCs was also significant (44840 ± 4807 mm^2^ versus 33681 ± 5548 mm^2^, *P* < 0.05; Fig. 6j). These imaging findings were further reinforced by immunohistochemistry staining of CD31 (Fig. 6k–n) and von Willebrand factor (Supplementary Fig. 6) in the peri-infarct area.

### Underlying Mechanism of Beneficial Biological Effects of MSCs Following ILK Overexpression

**Figure 7:**
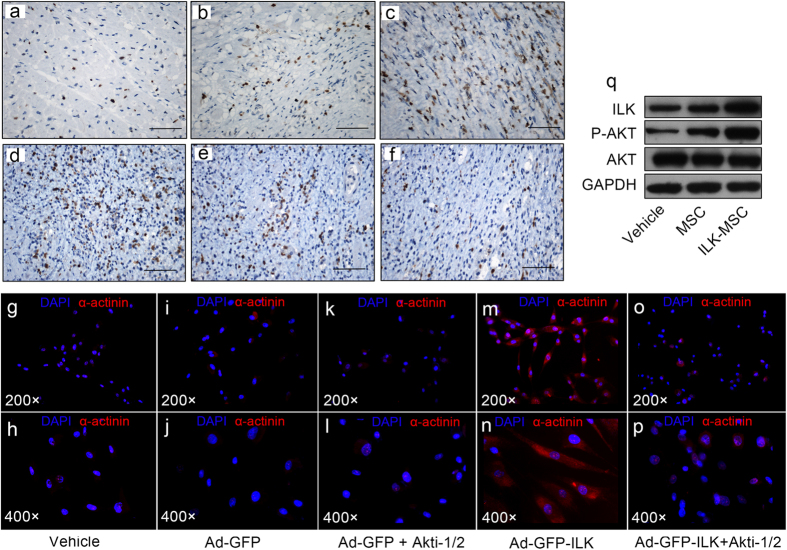
Underlying mechanism of beneficial biological effects of MSCs following ILK overexpression at cellular and molecular levels. (**a–f**) Immunohistochemistry staining of Ki67 (**a–c**) and Caspase 3 (**d–f**) in peri-infarct zones of hearts from vehicle- (**a,d**), vector-MSC- (**b,e**) and ILK-MSC-treated (**c,f**) minipigs at 15-day follow-up, indicating higher rates of cell proliferation and lower rates of apoptosis in ILK-MSC-treated animals (**c,f**). (**g–p**) Immunofluorescence assay stained for α-actinin showed that ILK overexpression promoted the differentiation of MSCs to cardiomyocyte-like cells *in vitro,* which is AKT-dependent. Red: α-actinin. Blue: DAPI. (**q**) Western blot analysis of the effects of ILK overexpression on the phosphorylation of AKT in MSCs. Scale bars in (**a–f**), 10 μm.

The results aforementioned showed the superiority of ILK-MSCs over vector-MSCs in the context of improving LV function through enhanced homing ability coupled with decreased infarct size and increased myocardium perfusion following myocardial infarction. Numerous mechanisms could account for these findings. In addition to the improved capillary density (improved angiogenesis), significantly higher rates of cell proliferation and lower rates of apoptosis were also noted in the peri-infarct area (Fig. 7a–f), which might be attributable to the paracrine effect of implanted ILK-MSCs, as demonstrated in our previous study that expression of matrix metallopeptidase (MMP)-9 and MMP-2 were significantly increased, while tissue inhibitors of metalloproteinase (TIMP)-1, TIMP-2, connective tissue growth factor (CTGF), and type I (Col1a1) and type III collagen (Col3a1) were significantly reduced in cardiac fibroblasts cultured under ILK-MSC conditioned medium^32^. Moreover, *in vitro* study revealed that ILK overexpression promoted the differentiation of MSCs into cardiomyocyte-like cells (Fig. 7g–p; Supplementary Fig. 7). At molecular level, significant inductions of phosphorylated AKT following ILK overexpression was observed by Western blotting analyses, indicating the involvement of ILK-AKT pathway (Fig. 7q).

## Discussion

**Figure 8:**
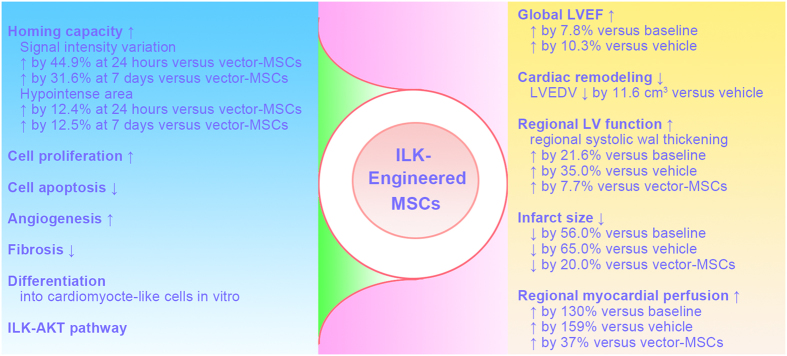
Overview of the beneficial effect (right column) of allogeneic ILK-MSCs and the potential mechanisms (left column) following intracoronary transplantation in porcine myocardial infarction.

Our preclinical study addresses for the first time the effect of ILK-MSCs therapy on global and regional cardiac function after acute ST-segment elevated MI induced by a transient coronary occlusion based on cardiac catheterization followed by reperfusion in swine. We visually tracked MSCs *in vivo* using the only viable approach—the combination of superparamagnetic iron oxide and MRI. Homing of MSCs to the infarct myocardium was significantly augmented following ILK overexpression, which was visually documented by MRI and confirmed by histological staining. Intracoronary infusion of ILK-MSCs to the infarct-related coronary artery significantly improves global LVEF and reverses cardiac remodeling compared with vehicles (or baseline), which of note was not achieved following vector-MSC treatment. Compared with vector-MSCs, ILK-MSCs significantly improved regional LV contractile function, substantially reduced scar size, cell apoptosis, and increased regional perfusion and cell proliferation. The main results of our study were shown in Fig. 8.

The necessity to augment the homing ability of stem cells arises due to paradox of selecting timing of cell transfer. Myocardial interstitial edema persists for at least one week after revascularization and the effect of transplantation at this time window might be limited^33^,^34^. However, implantation after 5 days following reperfusion exhibited limited homing efficacy^35^. Advantageously in our study, we initially engineered MSCs with ILK to enhance the homing and regenerative capacity of MSCs, which has been demonstrated in CPCs and EPCs^13^,^28^, and conducted cell transplantation 7 days after model establishment. Another superiority of our present study over other studies, including our prior work using open-chest model of MI^36^ was that we adopted catheter-based technique to induce MI, which avoided surgical wound on body surface that might comprise the homing capacity of MSCs to infarcted myocardium. Indeed, cells homed to jeopardised myocardium, as indicated by hypointense area and signal intensity variation recorded from T2*-weighted images and histological staining, were significantly higher following ILK-MSCs administration than vector-MSCs from 24 hours to 7 days. Interestingly, hypointense area observed at 15 days was lower in genetically ILK-MSCs compared with vector-MSCs, and even reduced by over 60% when compared with that at 7 days. A higher proliferative ability of MSCs after ILK overexpression could account for this seemingly contradictory result from MRI, given the proproliferative nature of ILK *in vitro* aforementioned in our study and other *in vivo* data^37^,^38^. In line with previous study, dilutional loss of iron occurred following cell proliferation^30^, leading to gradually diminishing signal void *in vivo*. Most importantly, our histological staining results validated this hypothesis by showing still significantly higher number of GFP- and iron-positive cells, while lower iron content per cell at 15 days in ILK-MSCs-treated myocardium, which could go beyond the detection threshold of MRI.

The synergic effect of MSCs and ILK in ILK-MSCs could be clued from preclinical studies using MSCs modified with AKT, one of the downstream effectors of ILK which was indeed activated in our study following ILK-MSCs implantation. Abeel and colleagues initially revealed that intramyocardial injection of MSCs overexpressing AKT inhibited the process of cardiac remodeling and normalized systolic cardiac function in rat myocardial infarction^27^. *ILK* gene offers potential advantages compared with *AKT* gene therapy based on its AKT-independent effects. For instance, transgenic mice with cardiac-specific overexpression of *ILK*^*R211A*^, a gain-of-function variant of *ILK*, exhibited increased baseline LV global systolic and diastolic functions through an initially discovered SERCA-2a/PLN mechanism^39^. *In vitro* transduction of human cardiomyocytes derived from induced pluripotent stem cells (iPSC-CMs) with *ILK*^*R211A*^ or wild type *ILK* (*ILK*^*WT*^) also caused a significant reduction in doxorubicin -induced apoptosis, which was not abrogated by the PI3K inhibitor LY294002 (indicating AKT-independent) or even by ILK kinase inhibition, revealing a scaffolding function of ILK that minimally involves CamKII, SERCA-2a, PLN and GSK-3β^39^. More straightforwardly, our present study further reinforced this overwhelming effect of ILK-MSCs, evidenced by sizable reduction in infarct size and fibrosis content, profound improvement in regional perfusion and vessel density when compared with vector-MSCs. It proved to be robust to reprogram MSCs with genetically modifying technique, fully exerting the antifibrotic and proproliferative properties^17^,^40^, and cardiomyogenesis potential^15^ of ILK in addition to the favorable characteristics of MSCs, and thus leading to significant recovery of impaired left ventricular function. In our study, ILK-MSCs treatment amounted to a 7.8% increment of LVEF compared with baseline, and reached by 10.3% when compared with vehicle in which LVEF progressively deteriorated and reduced by 2.5% at 22 days post infarction partially due to paucity of optimized pharmacological therapy such as angiotensin-converting enzyme inhibitor and beta-blockers^41^. A 7.8% gain of LVEF 15 days post-injection in our study mirrored those achieved with surgical-guided intramyocardial transplantation of MSCs in chronic ischemic cardiomyopathy, which however, were obtained from a dramatically longer-term follow-up^6^,^7^. Moreover, transendocardial delivery of MSCs for treating ischemic cardiomyopathy failed to detect the favorable effect on global LVEF in two clinical trials and one preclinical study published very recently^4^,^5^,^41^, and intravenous injection of human MSC yielded a profoundly lower increment in LVEF, with 2.6% gain at 3 months and 5.2% at 12 months^8^. Collectively, with regard to LV contractile performance, ILK-MSCs through intracoronary infusion outperformed unmodified MSCs even when intramyocardial delivered, which has great clinical implication for cell therapy in post-MI patients.

### Limitations

Several limitations should still be acknowledged in our study. First, the duration of follow-up in our study was relatively short. We selected a 15-day follow-up based on hypointense signals from the MRI-based *in vivo* tracking of iron-labeled MSCs. A dramatic reduction was observed at 15 days, which could be attributable to increased cell apoptosis at first glance. However, final histological examinations ruled this possibility out, and favorably, an augmented improvement of global and regional cardiac contractile function was achieved. Our study did not show that ILK-MSCs significantly outperformed vector-MSCs with regard to global LV function from direct comparisons; however, a trend toward advantage was seen from their comparisons with vehicles. A further improved benefit might be expected had the follow-up been longer and sample size been larger, since regional LV function was significantly improved and scar size reduced in ILK-MSCs than vector-MSCs. Second, the favorable effect of ILK-MSCs seen in our study could not be directly translated to clinical practice, because animals in our study did not receive optimized pharmacological therapies well established to inhibit myocardial remodeling and improve cardiac function post-MI, thus whether an add-on effect could be obtained on the basis of drug therapy is uncertain and needs confirmation in further studies. Third, our study was not designed to uncover the mechanism underlying the effect of ILK-MSCs. Therefore, a definite conclusion that ILK-MSCs transdifferentiated to cardiomyocytes *in vivo*^42^,^43^, promoted paracrine effects in ischemic tissue^44^ or enhanced proliferation of endogenous cardiac stem cells^45^ could not be reached, however, combined effects are more likely.

## Methods

Chinese experimental minipigs were obtained from Jiangsu Academy of Agricultural Sciences (Nanjing, China). Animal studies were approved by the Animal Ethics Committee of Nanjing University and were in compliance with the Chinese National Regulations on the Use of Experimental Animals.

### Isolation and culture of MSCs

The isolation and culture of MSCs were performed as in reference^46^ and detailed in Supplementary Materials.

### Recombinant viral vectors preparation and transduction

The ILK gene sequence was amplified by polymerase chain reaction (PCR) from a pUSEamp-ILK plasmid (a kind gift from professor Hyo-soo Kim from Seoul National University, South Korea), and the specific primer sequences were -ATC GAG TAC TAT GGA CGA CAT TTT C- and -GGG CCT CGA GCT ACT TGT CCT GCA T-. Recombinant adenoviral vectors were produced by GenScript (Nanjing, China). The methods were detailed in Supplementary Materials and Reference^17^.

### Assessment of cell viability, migration, proliferative ability, and apoptosis

Cell viability was evaluated by 3-(4,5-dimethylthiazol-2-yl)-2,5-diphenyltetrazolium (MTT, sigma) assay and measured spectrophotometrically at wavelength of 570 nm. Cell migration was assayed with 8-μm-pore size Transwell migration chambers as previously described. DNA synthesis and cell proliferation was assessed with 5-ethynyl-2´-deoxyuridine (EdU) assay and EdU-positive cells counted in 10 random × 400 fields and averaged, each experiment was performed in triplicate. Cell cycle analysis was performed using Propidium iodide staining followed by flow cytometric analysis, and apoptosis cell rate was estimated^28^.

### *In vitro* labeling of MSCs with ferumoxytol

The day after the last subculturing passage, swine MSCs were incubated with increasing concentrations of ferumoxytol (0, 10, 25, 50, 100, and 200 μg/ml) in the presence of poly-L-lysine (PLL) for different incubation periods (6, or 12, or 24, or 48 hours). Labeling efficiency was determined by Prussian blue staining, colorimetric ferrozine assay and transmission electron microscopy (TEM) study.

### Induction of myocardial infarction

Twenty minipigs were included. A guiding catheter was advanced to the left coronary artery through femoral artery puncture. After coronary angiogram, a coronary angioplasty balloon catheter was advanced and the balloon was placed in the left anterior descending artery (LAD) distal to the second diagonal branch and preconditioned for about 30 minutes followed by a 90-min occlusion, and then followed by reperfusion. Acute MI model was successfully established when substantial ST-segment elevation was observed from electrocardiography monitoring, and confirmed by images from late gadolinium enhancement MRI scanning.

### Intracoronary MSCs Delivery

Six to eight days after MI, an over-the-wire balloon catheter was advanced via a guiding catheter at the site of the previous blockage in the infarct-related artery. A 5-minute balloon inflation was performed to stop coronary flow beyond the balloon and to increase microvascular permeability. Vehicles (9 mL of PBS) or cells (50 million vector-MSCs or ILK-MSCs suspended in 9 mL of PBS) were infused over 9 minutes in three boluses during the 3-minute balloon inflation period and interrupted by 3 minutes of reflow by deflating the balloon.

### MR Imaging

See Supplementary Materials.

### Statistical analysis

Continuous variables are presented as mean ± SD or mean ± SEM as indicated. Homogeneity of variance was evaluated by Levene’s test. Differences between two groups were tested using independent samples t-test, whereas differences between three groups were compared with one-way analysis of variance followed by LSD test. Comparisons of changes from baseline within groups were performed using a paired Student t test. Comparison of changes in MRI variables between groups was conducted with analysis of covariance regarding each baseline variable as a covariate. All hypothesis tests were 2 sided, and a *P* value < 0.05 was considered statistically significant. All statistical analyses were performed with SPSS 17.0 software.

## Additional Information

**How to cite this article**: Mu, D. *et al.* Intracoronary Transplantation of Mesenchymal Stem Cells with Overexpressed Integrin-Linked Kinase Improves Cardiac Function in Porcine Myocardial Infarction. *Sci. Rep.* **6**, 19155; doi: 10.1038/srep19155 (2016).

## Supplementary Material

Supplementary Information
