# Supplementary material for: Intracoronary Transplantation of Mesenchymal Stem Cells with Overexpressed Integrin-Linked Kinase Improves Cardiac Function in Porcine Myocardial Infarction
Source: Sci Rep. 2016 Jan 11;6:19155. doi: 10.1038/srep19155 (PMC4707493; doi:10.1038/srep19155)
Supplement: Supplementary Information [file srep19155-s1.doc]

**Supplementary Materials and Methods**

# Intracoronary Transplantation of Mesenchymal Stem Cells with Overexpressed Integrin-Linked Kinase Improves Cardiac Function in Porcine Myocardial Infarction

Dan Mu,^1,2^,* Xin-Lin Zhang,^1^,* Jun Xie,^1^ Hui-Hua Yuan,^1^ Kun Wang,^1^ Wei Huang,^1^ Guan-Nan Li,^1^ Jian-Rong Lu,^1^ Li-Juan Mao,^1^ Lian Wang,^1^ Le Cheng,^2^ Xiao-Li Mai,^2^ Jun Yang,^3^ Chuan-Shuai Tian,^2^ Li-Na Kang,^1^ Rong Gu,^1^ Bin Zhu,^2^† Biao Xu,^1^†

Chinese experimental minipigs were obtained from Jiangsu Academy of Agricultural Sciences (Nanjing, China). Animal studies were approved by the Animal Ethics Committee of Nanjing University and were in compliance with the Chinese National Regulations on the Use of Experimental Animals.

**Isolation and culture of MSCs**

Swine bone marrow-derived cells were flushed out from iliac crest or femurs with culture medium consisting on DMEM (low glucose, Invitrogen) supplemented with 10% fetal bovine serum (FBS, Invitrogen) and 1% antibiotic-penicillin and streptomycin solution (Invitrogen). Mononuclear cells were isolated using Ficoll-Paque density gradient (GE Healthcare) centrifugation (1). After 48 hours incubation, nonadherent cells were discarded, and adherent cells were thoroughly washed twice with PBS. Thereafter, the culture medium was regularly changed every 3 to 4 days. About 2 weeks later, these cells expanded and reached confluent, thus subculturing was employed after detachment with 0.25% trypsin solution containing 0.01% EDTA (Invitrogen). Subsequent passages were performed in similar fashion. Passage 3 to 6 MSCs in 70–80% confluent were used in the study. The cultured cells were labeled with antibodies against various cell surface markers, including CD34, CD90, CD29, CD44, and CD45, and analyzed by flow cytometry.

**Recombinant viral vectors preparation and transduction**

The ILK gene sequence was amplified by polymerase chain reaction (PCR) from a pUSEamp-ILK plasmid (a kind gift from professor Hyo-soo Kim from Seoul National University, South Korea), and the specific primer sequences were -ATC GAG TAC TAT GGA CGA CAT TTT C- and -GGG CCT CGA GCT ACT TGT CCT GCA T-. Recombinant adenoviral vectors were produced by GenScript (Nanjing, China). Briefly, the amplified ILK sequence was subcloned into pShuttle-IRES-hrGFP-1 to produce recombinant vector pShuttle-ILK-hrGFP. The resultant plasmid is linearized by digesting with restriction endonuclease PmeI and subsequently transformed into competent BJ5183 cells. After homologous recombination, pAd-ILK recombinants are selected for kanamycin resistance and confirmed by restriction endonuclease analyses with PacI (2). The viral vectors were packaged by transfecting human embryonic kidney 293 cells.

For gene modification, MSCs cultured to 60% confluence were transfected with purified adenoviral vector at a multiplicity of infection (MOI) of 5. After incubation for 12 hours, the adenoviral media was removed, and replaced with fresh complete medium. The adenovirus without ILK cDNA and vehicle PBS served as controls. The transfection efficiency was evaluated via fluorescence microscopy and flow cytometry 48 hours after the infection, as well as western blot analysis and quantitative real-time polymerase chain reaction to assess ILK expression.

**Quantitative real-time polymerase chain reaction**

Real-time PCR was performed in a total volume of 10 μl with 5 μl of 2×SYBR Premix Ex Taq (TaKaRa), 0.2 μl of 50×Rox dye (TaKaRa), 0.4 μl of each primer (4 pmoles each),

1.0 μl of cDNA and 3.0 μl ddH2O and was conducted in triplicate for each sample in an ABI StepOne Plus Real-Time PCR system. Primers were designed using the sequence within the junction of two exons using the online software Primer3 (<http://frodo.wi.mit.edu/primer3/input.htm15>), GAPDH was used as the endogenous reference to normalize gene expression, and the 2^-△△CT^ method was used to determine the differences in mRNA content between samples. The primers used were GAPDH forward -GGG CAT GAA CCA TGA GAA GT-, reverse -GTC TTC TGG GTG GCA GTG AT-; and ILK forward -TTT GCA GTG CTT CTG TGG GAA-, reverse -CTA CTT GTC CTG CAT CTT CTC-.

**Western blotting**

Western blotting analysis was performed and expression of proteins and phosphorylated protein levels was normalized to GAPDH. Proteins were separated by 10% SDS-PAGE and transferred to a polyvinylidene diflouride (PVDF) membrane (Millipore Corporation, USA), which was then blocked with 5% non-fat dried milk for 2 h with gentle shaking. Subsequently, the membrane was incubated with 5% bovine serum albumin (Roche) together with specific antibody overnight at 4℃. Antibody dilutions were Akt (1:500; BD Pharmingen), p-Akt (Ser473; 1:500; BD Pharmingen), eNOS (1:500; CST), p-eNOS (1:500; CST), GAPDH (1:5000; Bioworld), ILK (1:1000; BD Pharmingen).

**Assessment of cell viability, migration, proliferative ability, and apoptosis**

Cell viability was evaluated by 3-(4,5-dimethylthiazol-2-yl)-2,5-diphenyltetrazolium (MTT, sigma) assay and measured spectrophotometrically at wavelength of 570 nm. Cell migration was assayed with 8-μm-pore size Transwell migration chambers as previously described (3). Briefly, cell migration was allowed to proceed for 24 hours at 37°C, and cells that migrated were stained with 0.1% (w/v) crystal violet for 20 minutes and washed with PBS. At least 10 random ×200 fields were counted for each experimental condition. DNA synthesis and cell proliferation was assessed with 5-ethynyl-2´-deoxyuridine (EdU) assay and EdU-positive cells counted in 10 random ×400 fields and averaged, each experiment was performed in triplicate. Cell cycle analysis was performed using Propidium iodide staining followed by flow cytometric analysis, and apoptosis cell rate was estimated.

***In vitro* labeling of MSCS with feumoxytol**

The day after the last subculturing passage, swine MSCs were incubated with increasing concentrations of feumoxytol (0, 10, 25, 50, 100, and 200 μg/ml, from University of Miami Miller School of Medicine) in ddH_2_0 in the presence of poly-L-lysine (PLL, Sigma) for different incubation periods (6, or 12, or 24, or 48 hours). Feumoxytol solutions were prepared at a concentration of 5 mg/ml, and PLL from stock solution was added (final Fe/PLL ratios were 1:0.05) and allowed to mix for 60 min. Double distilled H_2_O without feumoxytol was added to sister cultures of MSCs as untreated controls. At the end of the incubation period, cells were collected and washed twice in PBS. The labeling efficiency was determined by Prussian blue staining, colorimetric ferrozine assay and transmission electron microscopy (TEM) study. Cell proliferation, migration, apoptosis and cell cycles were also analyzed in each group.

**Prussian blue staining**

After incubation with feumoxytol, cells were washed 2 times to remove excess iron. Cells were fixed with 4% paraformaldehyde for 20 minutes, washed twice, and incubated for 30 minutes with Pearls’ reagent (4% Potassium ferrocyanide/12%HCl, 50:50 vol/vol). Cultures were then washed once in PBS and samples were observed in light microscopy.

**Colorimetric ferrozine assay**

A total of 1.5 million cells were harvested, washed and volume to 50 μl, and mixed with the iron-releasing reagent (a fresh solution of 11 μl of 1.4 M HCl). These mixtures were incubated for 20 minutes at 95^o^C. After the mixture had cooled to room temperature, cells were centrifuged and 45 μl supernatant was removed, and then the iron-detection reagent (18 μl of ascorbic acid, 18 μl of ferrozine, and 36 μl of ammonium acetate) was added in sequence to each sample. The absorbance of the sample solution was measured at 562 nm on a microplate reader. Each experiment was repeated 3 times.

**Transmission electron microscopy (TEM) study**

Cells were prepared in PBS and centrifuged at 4000 rpm for 20 minutes. The supernatant was gently aspirated and the pellet was collected for electron microscopy processing. Pellets were fixed with 3% glutaraldehyde at 4^o^C for 4 hours, washed with PBS for 3 times and then incubated in 1% osmium tetroxide for 2 hours. The samples were then processed through graded alcohols for dehydration, propylene oxide infiltration at room temperature. The pellets were embedded in epoxy resin, sectioned at 70 nm and stained with 1% uranyl acetate and lead citrate, then scanned with an H600-IV transmission electron microscope (Hitachi, Japan).

**Induction of myocardial infarction**

Chinese experimental minipigs (20 ± 5 kg) were pre-anesthetized with an intramuscular injection of ketamine hydrochloride (12 mg/kg), droperidol (0.5 mg/kg), and diazepam (1 mg/kg), and maintained under general anesthesia by a continuous infusion of propofol (5 mg/kg/h) throughout the procedure. The animals were intubated and ventilated with 60% O_2_. Electrocardiograms and pulse oximeter measurements were continuously monitored. A guiding catheter was advanced to the left coronary artery through femoral artery puncture. After coronary angiogram, a coronary angioplasty balloon catheter was advanced and the balloon was placed in the left anterior descending artery (LAD) distal to the second diagonal branch and preconditioned for about 30 minutes followed by a 90-min occlusion, and then followed by reperfusion (4). In case of malignant arrhythmia, resuscitation via electrical cardioversion was applied immediately with continuous chest compression. Intravenous injections of epinephrine, atropine, and lidocaine and sodium bicarbonate *etc.* were administered in the rescuing process. Acute myocardial infarction (MI) model was successfully established when substantial ST-segment elevation was observed from electrocardiography monitoring. Five healthy non-infarcted minipigs were included, and their cardiac functions were used as references.

**Intracoronary MSC Delivery**

Six to eight days after MI, minipigs were anesthetized as described above. The other-side femoral artery of was cannulated. An over-the-wire balloon catheter was advanced via a guiding catheter at the site of the previous blockage in the infarct-related artery. A 5-minute balloon inflation was performed to stop coronary flow beyond the balloon and to increase microvascular permeability. Vehicles (9 mL of phosphate-buffered saline (PBS)) or cells (50 million vector-MSCs or ILK-MSCs suspended in 9 mL of PBS) were infused over 9 minutes in three boluses during the 3-minute balloon inflation period and interrupted by 3 minutes of reflow by deflating the balloon.

**MR Imaging**

Cardiac magnetic resonance imaging were performed on a 1.5-T scanner (Philips Achieva, Cleveland, OH) using an 8-element phased-array surface coil with ECG gating and ventilator breath-holding. Animals were imaged within the first 24 hours following intracoronary MSCs injection, to assess the migration of iron-labeled MSCs in vivo, as well as at 7-day and 15-day follow-up. T2*-weighted gradient-echo scans were used with a repetition time (TR) of 35 ms, an echo time (TE) of 14 ms, a matrix of 192 × 192, a field of view (FOV) of 240 mm, a flip angle (FA) of 60°, a band width (BW) of 32 kHz, a slice thickness of 4.0 mm with a 0.2-mm gap. Iron particles were detected as a signal void using this sequence. Because heart rates of minipigs following myocardium infarction were fast, and the resulting considerable motion artifacts of apex area could influence the identification of hypointense area in short axis view, we chose the 4-chamber view which covered the whole left ventricular wall to measure the hypointense area and signal intensity value. We also referred to images from short axis view to guide our analysis. The hypointense area was calculated as the sum of the area of visually determined signal void in all slices. We defined signal intensity variation as the difference value of signal intensity between normal myocardium and infarcted myocardium. Signal intensity was measured in at least 3 consecutive 4-chamber long-axis slices. All measurements were performed by investigators who were blinded to the allocation of injections.

Cardiac performance was evaluated at baseline and 15 days after PBS or MSCs transplantation to determine the efficacy of implantation. Cine MRI acquisitions were performed using balanced fast field echo (BFFE) sequence in the long-axis (2-chamber and 4-chamber views) and short-axis orientation with following parameters: 3.5-ms TR, 1.7-ms TE, 45° FA, 320 × 320 mm FOV, and 8-mm slice thickness. Analyses of left ventricular ejection fraction (LVEF), left ventricular end-diastole volume (LVEDV) and wall motion were achieved with the Cardiac Explorer software package. In each animal, 8 to 10 short-axis sections were obtained, covering the entire left ventricle. Infarcted myocardial segments were visually positioned from matched delayed contrast-enhanced images, and systolic wall thickening was calculated on short-axis images as following formula to indicate regional contractile function: [(WES - WED)/ WED]*100, where WES indicated end-systolic wall thickness and WED indicated end-diastolic wall thickness. The value of the percentage systolic wall thickening was averaged in 3 consecutive short-axis slices.

Myocardial perfusion was evaluated with the first-pass perfusion imaging after bolus injection of 0.1 mmol/kg Gd-DTPA with a rate of 4.0 ml/s using a single-shot CE-FFE-T1 (Contrast-Enhanced Fast Field Echo) sequence. The parameters used were as follows: 4.8-ms TR, 1.23-ms TE, 20° FA, 247 × 230 mm FOV, 8-mm slice thickness, and 30 heart beats. Short-axis sections were positioned covering the infarcted area as identified with initial imaging, as well as the basal and LV blood pool. Time-intensity curves (TICs) were generated and the area under curves (AUC) was computed as parameter reflecting blood supply in certain region.

After first-pass perfusion, an additional 0.1 mmol/kg of Gd-DTPA was administered. Delayed contrast-enhanced imaging was performed 10 to 15 minutes later using a 2-dimensional segmented inversion recovery gradient-echo pulse sequence in continuous short-axis, 4-chamber and 2-chamber long axis views with full coverage of the LV to assess infarct size. The FA was 15°, with TR 5.1 ms, TE 2.5 ms, FOV 255 × 255 mm and slice thickness 8 mm. The inversion time was chosen to minimize the signal from normal myocardium. A semiautomatic approach was used for quantification of hyperenhanced myocardium: epicardial and endocardial contours were manually drawn, and region of interests (ROIs) were placed in hyperenhanced and normal myocardium by the build-in software. The areas of delayed enhancing myocardium were then automatically segmented by using a full-width at half-maximum algorithm. The threshold could be manually overridden only if necessary to exclude significant artifacts. Infarct size was calculated automatically as the ratio of area of myocardium with delayed enhancement to the area of LV myocardium in each slice from the short-axis delayed-enhancement images. Total infarct size was calculated by summation of all slice volumes of hyperenhancement and divided by slice number. Infarct size change was defined as the difference of the infarct size after cell transplantation to infarct size at baseline.

**Morphometry, tissue section preparation, and histological analysis**

The pigs were euthanized as aforementioned and received an intravenous bolus of 10% KCl (40 to 60 ml) 15 days post-transplantation, and heart samples were collected for histology analysis. The infarcted myocardium area was measured, as well as the thickness of infarct and normal myocardium, as previously described (5). Representative samples were selected from the infarct zone, peri-infarct zones, and remote zones. The myocardium was snap-frozen in liquid nitrogen and stored at -80°C, or fixed in 10% buffered neutral formalin at room temperature for 8-12 hours. The frozen blocks were embedded in OCT and sectioned into 5 μm for subsequent assays. The formalin-fixed tissue was embedded in paraffin wax and cut into 5 μm sections before use. All histological analyses were performed within the infarct border zone.

To determine whether ILK-MSC transplantation influences collagen fiber accumulation, fibrosis was analyzed by Masson’s Trichrome staining in peri-infarct zones. We randomly selected 5 fields per slice and calculated collagen volume fraction (CVF) as the ratio of Masson's Trichrome-stained collagen area to total myocardium area (6), which was measured with Image Pro Plus software (Media Cybernetics).

**Immunofluorescence and Immunohistochemistry**

Engraftment of MSCs in swine hearts was assessed with immunofluorescence from frozen sections and the percent of green fluorescent cells were counted. At least 10 sections of each heart were measured and averaged.

To detect whether ILK-engineered MSCs promoted cell proliferation in peri-infarct zones, immunohistochemical analysis was performed for Ki-67. Tissue sections were deparaffinized and subjected to antigen retrieval in 10 mmol/l sodium citrate (pH 6.0) in a microwave oven for 15 min. Endogenous peroxidase was blocked with 0.3% hydrogen peroxide for 15 min. After incubation in blocking buffer (goat serum) for 30 min, the sections were exposed to primary antibody (1:100 mouse anti-Ki-67, BD) overnight at 4^o^C. The slides were then washed in PBS for 5 min×3 and incubated with secondary antibody for 30 min. Incubation with the chromogen (3,3’-diaminobenzidine) was carried out for 10 min. The nuclei were counterstained with hematoxylin. Images were captured with an optical microscope (Olympus Bx50), and processed using Sigma scan software. The number of Ki67-positive cells was calculated in peri-infarct zones and was averaged over at least ten fields per specimen. Cell apoptosis was evaluated by performing immunohistochemical analysis of Caspase 3 using a Caspase 3 kit from KeyGen Company.

To measure microvessel density in the border area, frozen sections were stained with anti-mouse CD31 antibody (1:200, Zymed) and anti-von Willebrand factor (vWF) antibody (1:200, Abcam). The numbers of CD31-positive cells were determined by counting at least 10 random fields in each section in a blinded fashion at ×100 magnification. Three sections of each heart were measured and averaged.

**Supplementary Figures and Figure legends**

**
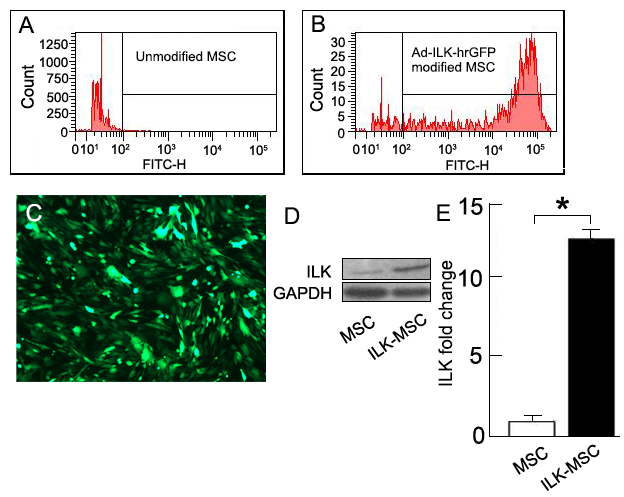
**

**Supplementary Figure 1. Engineering MSCs with Ad-ILK-hrGFP. (A to B)** Modification efficiency was 90.3% as measured by GFP expression with flow cytometry analysis. **(C)** Expression of hrGFP in the modified MSCs was confirmed by immunofluorescent assay. **(D to E)** ILK expression was confirmed by western blot analysis **(D)** and quantitative real-time polymerase chain reaction **(E)**. Data are mean ± SD. **P* < 0.05 between comparisons indicated by bracket.

**
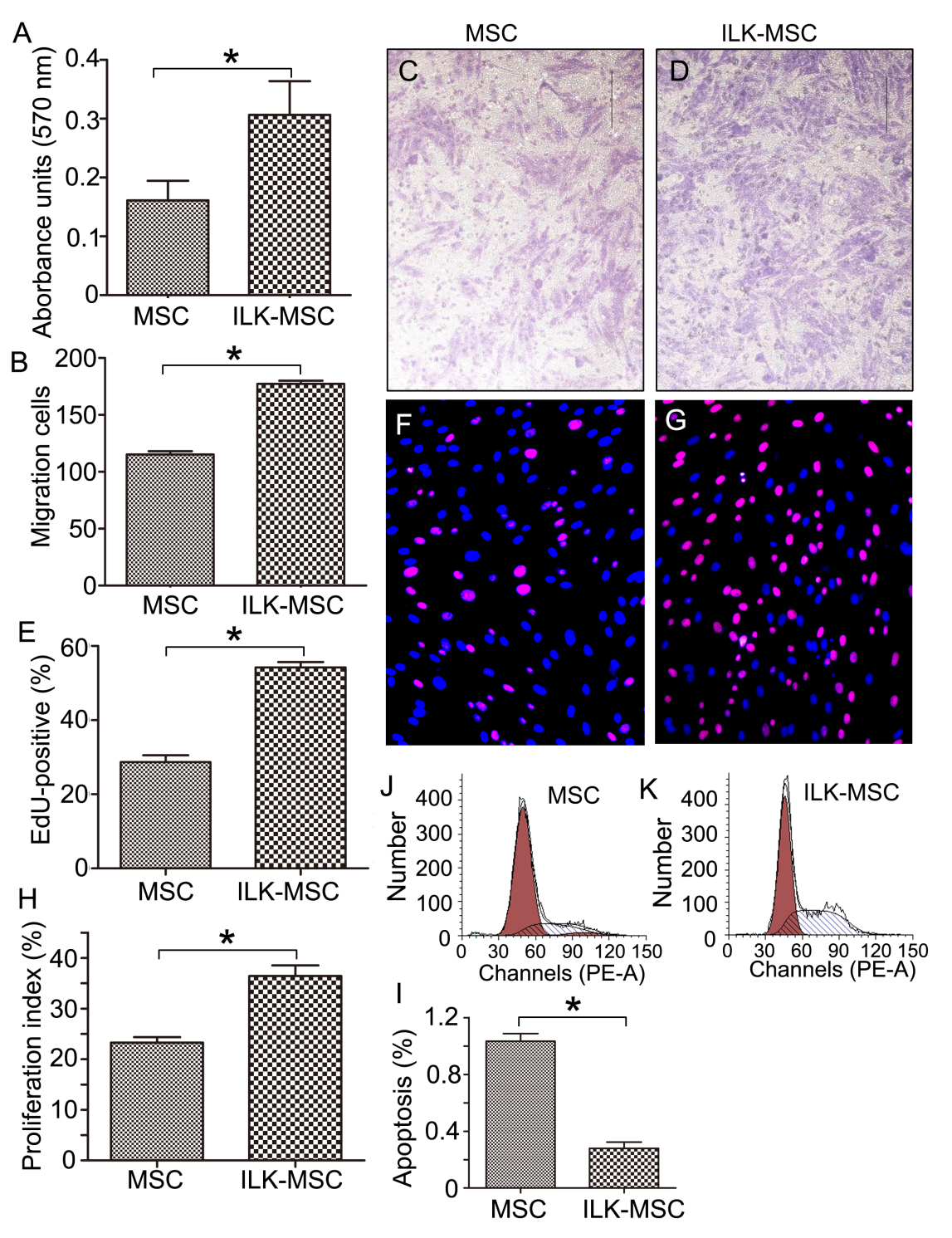
**

**Supplementary Figure 2. ILK overexpression in MSCs. (A)** ILK-engineered MSCs showed increased cell viability than vector-engineered MSCs, as confirmed by 3-(4,5-dimethylthiazol-2-yl)-2,5-diphenyltetrazolium assay. **(B to D)** *In vitro* migration of MSCs was enhanced following ILK modification as determined by transwell migration assay. **(E to G)** ILK-engineered MSCs had significantly higher level of proliferation evaluated with 5-ethynyl-2´-deoxyuridine assay. **(H to K)** Cell cycle analysis performed using Propidium iodide staining followed by flow cytometric analysis revealed a higher level of proliferation, lower level of apoptosis in ILK-engineered MSCs. Data are mean ± SD. **P* < 0.05 between comparisons indicated by bracket.

**
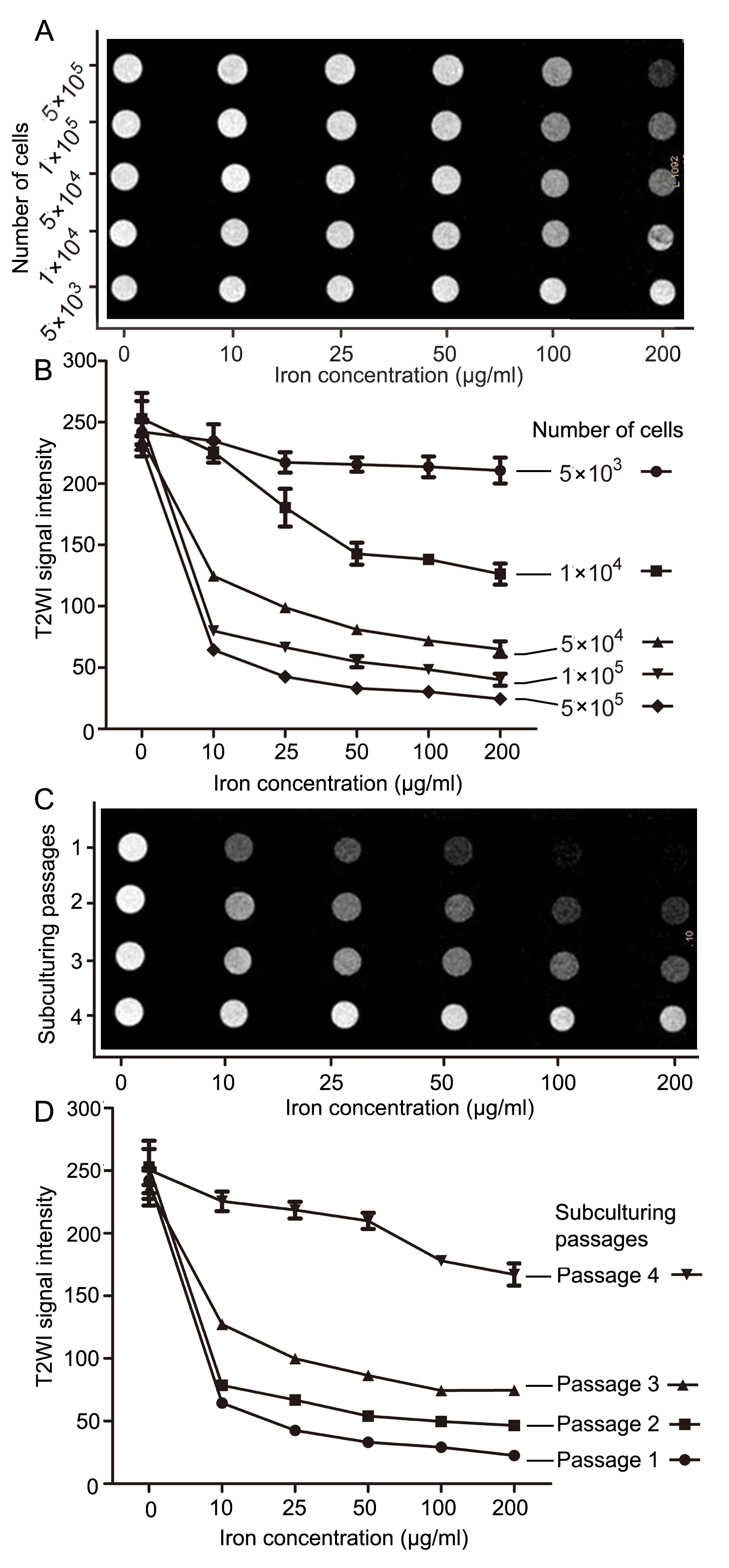
**

**Supplementary Figure 3. *In vitro* detection of iron-labeled MSCs with magnetic resonance imaging (MRI). (A to B)** T2-weighted MRI were performed using increasing number of MSCs (5 × 10^3^ to 5 × 10^5^) subcultured at increasing concentrations of ferumoxytol (0 to 200 μg/ml). When treating 1 × 10^4^ MSCs with 100 μg/ml ferumoxytol, the hypointense signals from T2-weighed images were distorted due to blooming effect. **(C to D)** T2-weighted MRI were performed using of MSCs from different subculturing passages (passage 1 to 4) subcultured at increasing concentrations of ferumoxytol (0 to 200 μg/ml). An inverse correlation was found between negative signal intensity and number of passages. Data are mean ± SD.

**
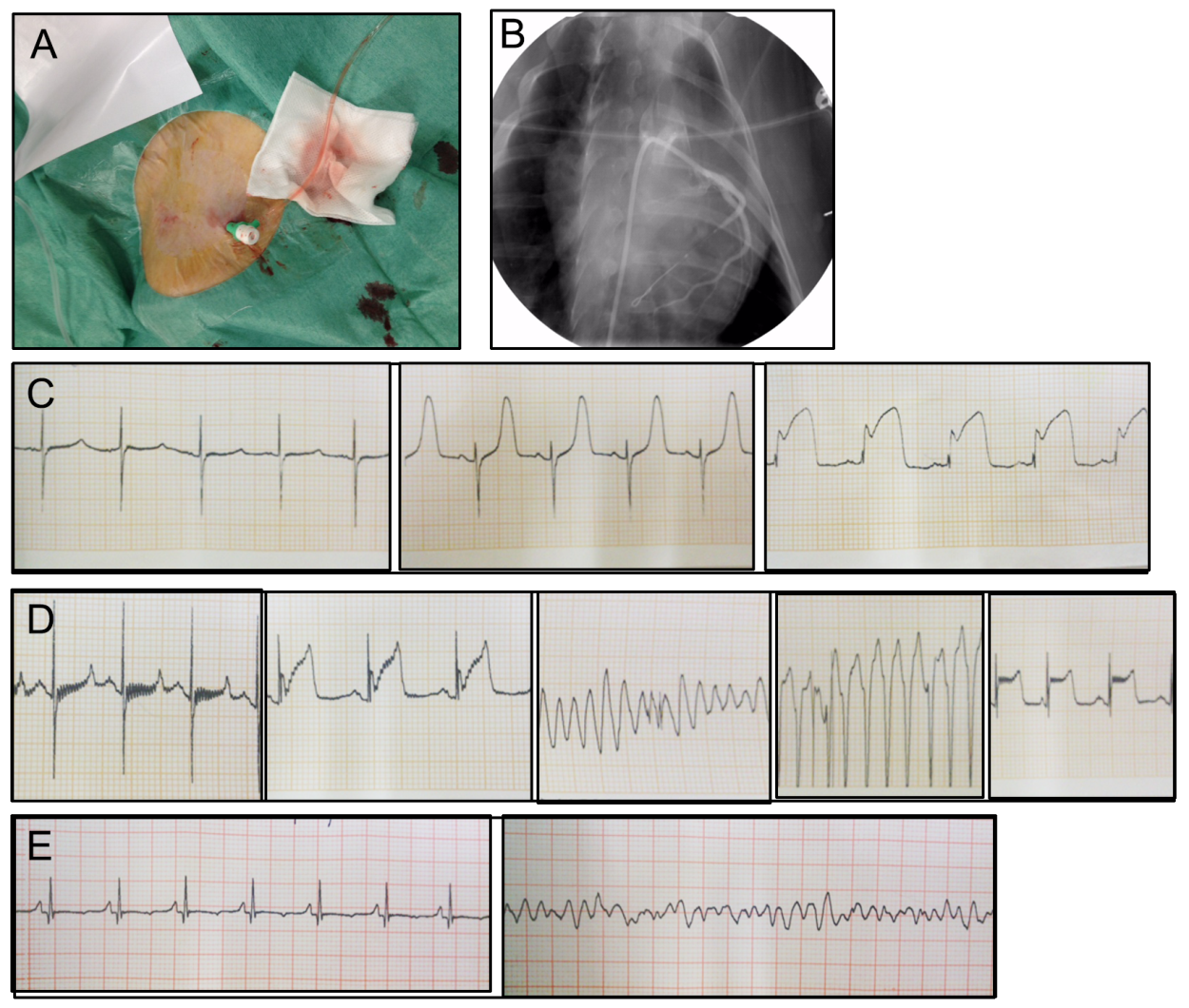
**

**Supplementary Figure 4. (A)** Procedure field. **(B)** Coronary arteriography after myocardial infarction (MI). **(C to E)** The electrocardiogram was performed before **(left)**, during **(middle)** and after **(right)** the induction of experimental MI. **(C)** MI model was smoothly established. **(D)** MI was successfully induced following repeated defibrillation and pharmacological therapy. **(E)** MI model was not established due to ventricular fibrillation given repeated defibrillation and pharmacological therapy, and animals died.

**
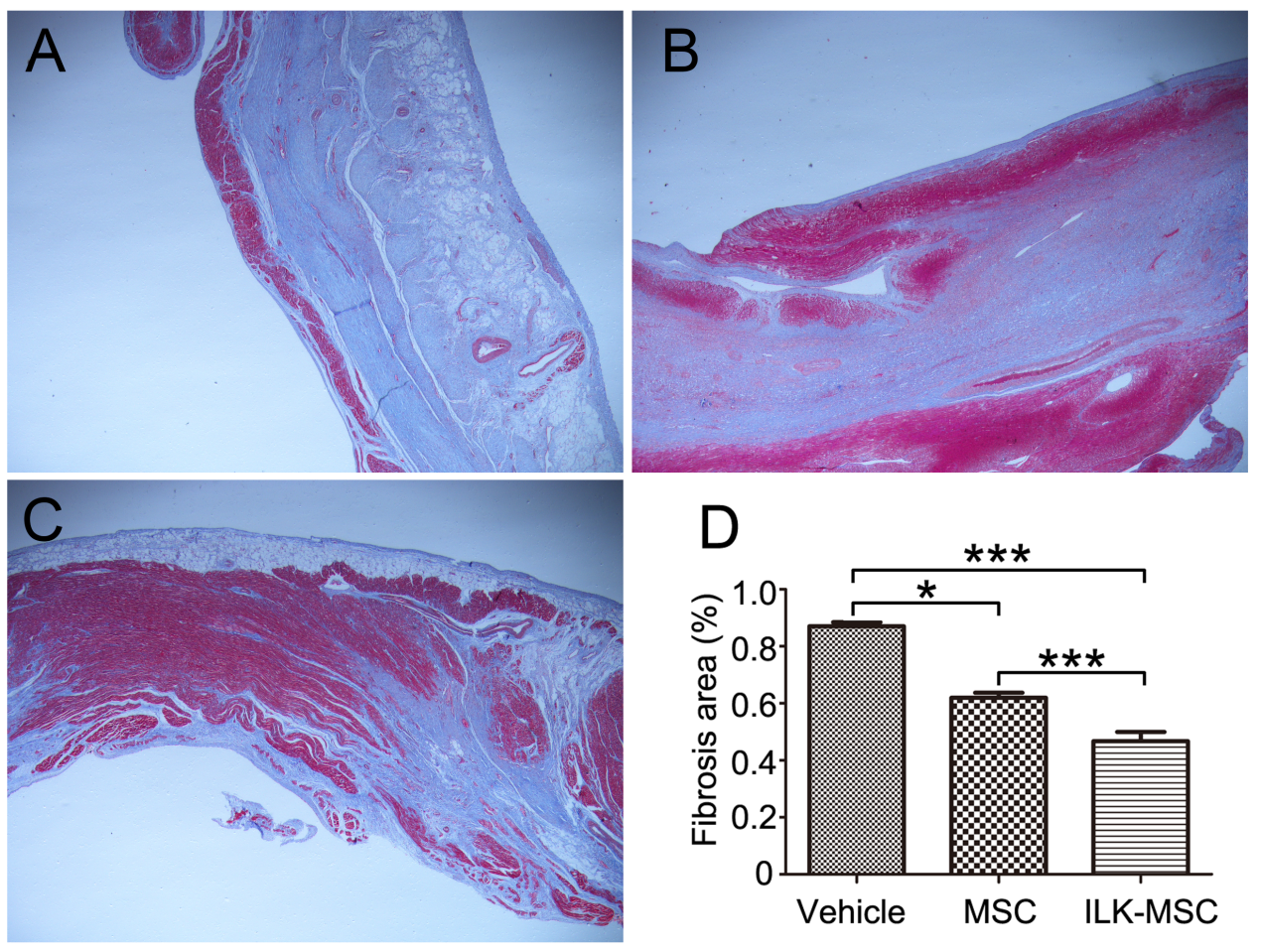
**

**Supplementary Figure 5. ILK-MSC transplantation decreased infarct scar size. (A to C)** Representative sections from the peri-infarcted wall of one vehicle-, one vector-MSC- and one ILK-MSC-treated minipigs at 15 days post-implantation stained with Masson’s trichrome. **(D)** Percent of fibrosis area at 15 days post-implantation. Data are mean ± SD. **P* < 0.05, ****P* < 0.001 between comparisons indicated by bracket.


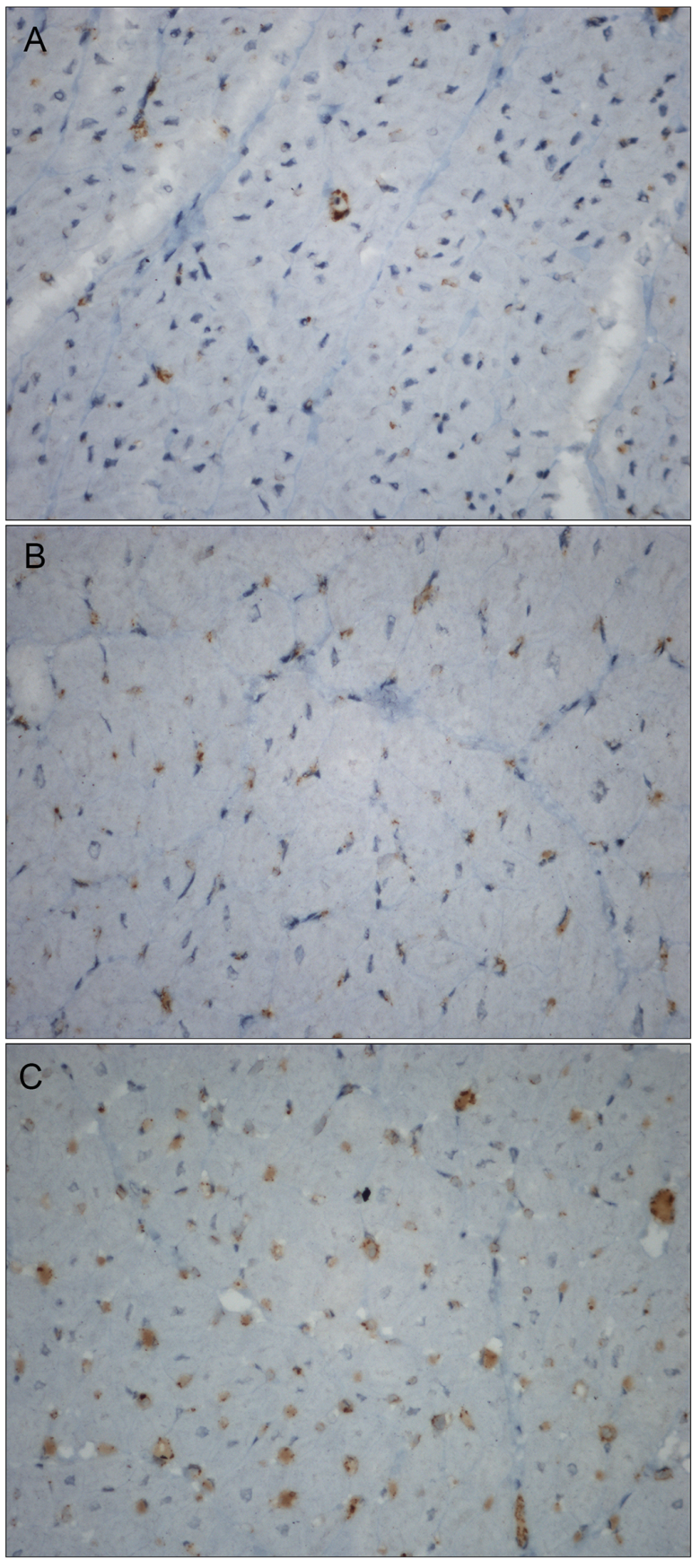


**Supplementary Figure 6. Immunohistochemistry staining of VWF in peri-infarct zones in one vehicle- (A), one vector-MSC- (B) and one ILK-MSC-treated (C) minipig.** These data confirmed an enhanced microvessel density in ILK-MSC treated animals.


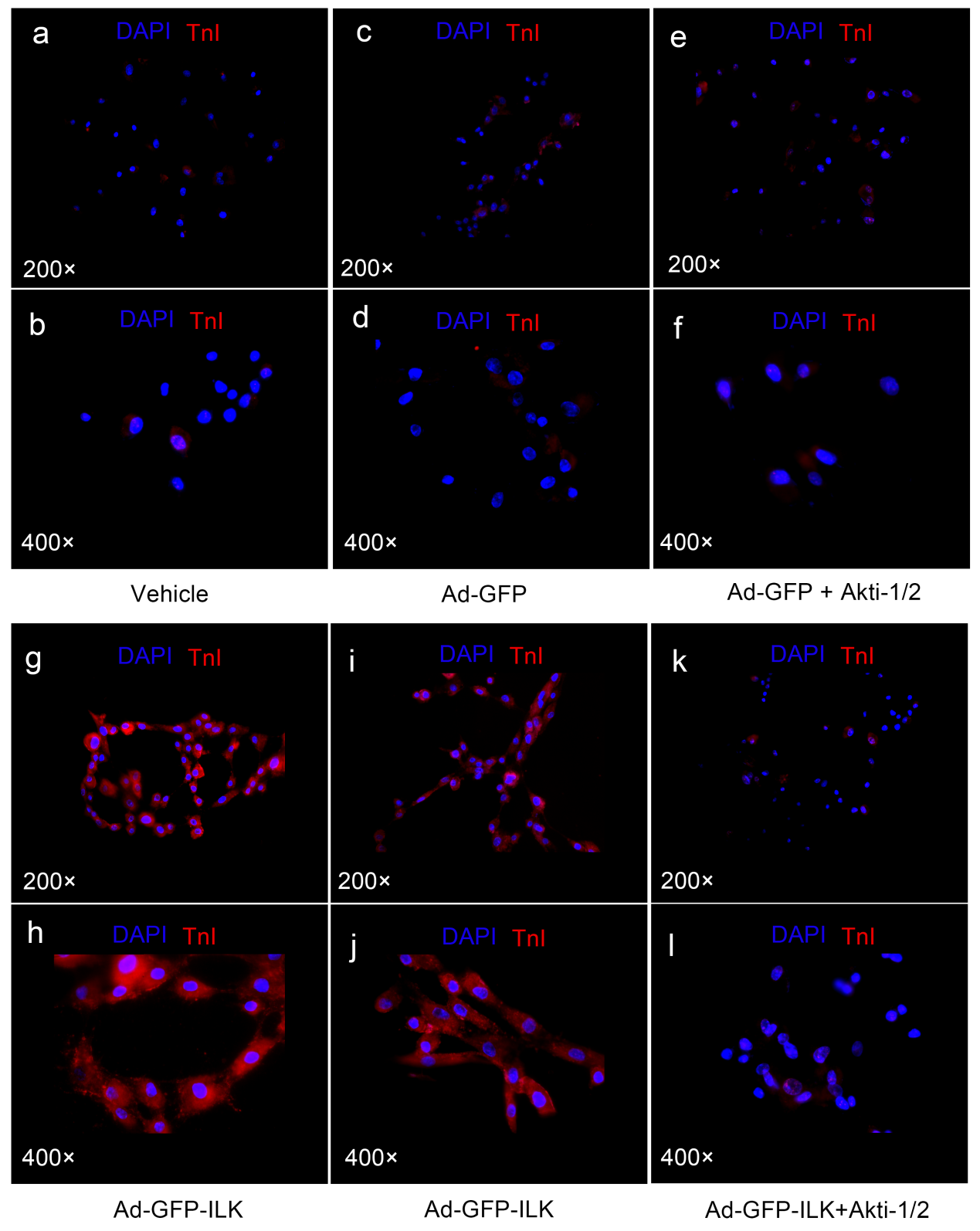


**Supplementary** Figure 7. Immunofluorescence assay stained for TNI showed that ILK overexpression promoted the differentiation of MSCs to cardiomyocyte-like cells in vitro, which is AKT-dependent. Red: TNI. Blue: DAPI.

**Supplementary** **References**

1. Williams AR, Trachtenberg B, Velazquez DL, et al. Intramyocardial stem cell injection in patients with ischemic cardiomyopathy: functional recovery and reverse remodeling. Circ Res 2011;108:792-6.

2. Luo J, Deng Z, Luo X, et al. A protocol for rapid generation of recombinant adenoviruses using the AdEasy system. Nature Protocols 2007;2:1236-1247.

3. Senger DR, Perruzzi CA, Streit M, Koteliansky VE, de Fougerolles AR, Detmar M. The alpha(1)beta(1) and alpha(2)beta(1) integrins provide critical support for vascular endothelial growth factor signaling, endothelial cell migration, and tumor angiogenesis. Am J Pathol 2002;160:195-204.

4. Qi CM, Ma GS, Liu NF, et al. Transplantation of magnetically labeled mesenchymal stem cells improves cardiac function in a swine myocardial infarction model. Chin Med J (Engl) 2008;121:544-50.

5. Ding L, Dong L, Chen X, et al. Increased expression of integrin-linked kinase attenuates left ventricular remodeling and improves cardiac function after myocardial infarction. Circulation 2009;120:764-73.

6. Barbone A, Oz MC, Burkhoff D, Holmes JW. Normalized diastolic properties after left ventricular assist result from reverse remodeling of chamber geometry. Circulation 2001;104:I229-32.
